# Rotavirus intestinal infection induces an oral mucosa cytokine response

**DOI:** 10.1371/journal.pone.0195314

**Published:** 2018-04-05

**Authors:** José Gómez-Rial, María José Curras-Tuala, Irene Rivero-Calle, Carmen Rodríguez-Tenreiro, Lorenzo Redondo-Collazo, Alberto Gómez-Carballa, Jacobo Pardo-Seco, Antonio Salas, Federico Martinón-Torres

**Affiliations:** 1 Grupo de Investigación en Genética, Vacunas, Infecciones y Pediatría (GENVIP), Hospital Clínico Universitario de Santiago de Compostela, Galicia, Spain; 2 Laboratorio de Inmunología, Servicio de Análisis Clínicos, Hospital Clínico Universitario de Santiago de Compostela, Galicia, Spain; 3 Translational Pediatrics and Infectious Diseases, Department of Pediatrics, Hospital Clínico Universitario de Santiago de Compostela, Galicia, Spain; 4 GenPoB Research Group, Instituto de Investigaciones Sanitarias (IDIS), Hospital Clínico Universitario de Santiago de Compostela, Galicia, Spain; 5 Unidade de Xenética, Departamento de Anatomía Patolóxica e Ciencias Forenses, Instituto de Ciencias Forenses, Facultade de Medicina, Universidade de Santiago de Compostela, Galicia, Spain; University of Liverpool, UNITED KINGDOM

## Abstract

**Introduction:**

Salivary glands are known immune effector sites and considered to be part of the whole mucosal immune system. The aim of the present study was to assess the salivary immune response to rotavirus (RV) infection through the analysis of the cytokine immune profile in saliva.

**Material and methods:**

A prospective comparative study of serial saliva samples from 27 RV-infected patients (sampled upon admission to the hospital during acute phase and at convalescence—i.e. at least three months after recovery) and 36 healthy controls was performed. Concentrations of 11 salivary cytokines (IFN-γ, IFN-α_2_, IL-1β, IL-6, IL-8, IL-10, IL-15, IL12_p70_, TNF-α, IFN-λ_1_, IL-22) were determined. Cytokine levels were compared between healthy controls acute infection and convalescence. The correlation between clinical data and salivary cytokine profile in infected children was assessed.

**Results:**

The salivary cytokine profile changes significantly in response to acute RV infection. In RV-infected patients, IL-22 levels were increased in the acute phase with respect to convalescence (*P*-value < 0.001). Comparisons between infected and control group showed significant differences in salivary IFN-α_2_, IL-1β, IL-6, IL-8, IL-10 and IL-22. Although acute-phase levels of IL-12, IL-10, IL-6 and IFN-γ showed nominal association with Vesikari’s severity, this trend did not reach statistical significance after multiple test adjustment.

**Conclusions:**

RV infection induces a host salivary immune response, indicating that immune mucosal response to RV infection is not confined to the intestinal mucosa. Our data point to a whole mucosal implication in the RV infection as a result of the integrative mucosal immune response, and suggest the salivary gland as effector site for RV infection.

## Introduction

Epithelial cells are more than mere physical barriers to infection; they also have a central role in immune responses and are key in tissue remodeling after healing [1]. Oral mucosa epithelial cells, located at the interface between the external and internal environment, are routinely exposed to large amounts of substances, including pathogens. Several studies have revealed that the oral epithelium–like all other epithelia-, is highly dynamic and displays a broad spectrum of activities related to immunity and host defense [2,3]. The major secretion associated with the oral mucosa epithelium is saliva, produced by the salivary glands and secreted to the oral cavity through the oral mucosa. Among the substances produced in saliva are cytokines [4], soluble regulating agents of immune responses, secreted by epithelial cells in response to diverse stimuli such as injury, infection or inflammation [5].

Saliva from the sublingual compartment has demonstrated to be an excellent non-invasive proxy for intestinal immune induction [6,7]. Induction of mucosal immunity in the intestinal Peyer’s patches, results in an effector response at distant sites such as the oral mucosa, functionally based on the compartmentalized mucosal immune system [7,8]. This fact has long been demonstrated with IgA and plasma cells [9–12], but, to the best of our knowledge, never before with the induction of an epithelial cytokine response. Until now, studies had only shown an oral epithelial cytokine response to local inflammation or infections of the mouth such as periodontitis [13–15], or in autoimmune diseases affecting oral mucosa such as Sjögren disease [16].

Here, we hypothesized that RV intestinal infection induces in the host a complete cytokine response at the oral mucosa epithelial cells, which can be measured in the sub-lingual compartment. If true, this would constitute supportive evidence to the suggestion that RV infection induces a whole mucosal immune response in locations as distant as the oral mucosa.

## Methods and material

### Patients and controls

A total of 27 RV infected patients admitted to hospital and 36 healthy children scheduled to receive RV vaccination were enrolled in this study. Written consent from parents was obtained for all subjects involved in this study. Approval for this project was obtained from the Ethics Committee for Clinical Research of Galicia before patients and healthy controls were recruited.

RV-infected patients were prospectively recruited at the Hospital Clínico Universitario of Santiago de Compostela (Spain) during the period 2013–2014, all of them hospitalized with acute gastroenteritis and with a positive RV antigen detected in stool. For the RV-infected group, demographic and clinical data were obtained, including detailed symptoms scores during the course of illness such as temperature, number of vomiting episodes per day and duration of vomiting, the severity of diarrhea (number of stools per day, duration of diarrhea and level of dehydration), and length of stay in hospital, as well as Vesikari’s severity score.

In the same period, 36 healthy children who attended to the infectology consultation for scheduled rotavirus vaccination were enrolled in the study.

### Samples

Saliva samples were collected at recruitment in acute phase and at convalescence (>90 days after infection) for RV-infected patients, and at recruitment (baseline pre-vaccination) for control group.

A sample from unstimulated sublingual saliva was obtained with oral swabs (*Whatman*) placed under the tongue for 5 min. The swabs were eluted in 0.4 ml of phosphate buffered saline (PBS), and then centrifuged at 800 g for 10 min to remove mucin and epithelial cells. Supernatants were stored at -30°C prior to analysis.

### Detection of cytokines in saliva samples

The cytokine detection assay was performed according to the instructions of the Milliplex Map human cytokine detection kit purchased from Millipore (Merck Millipore, Billerica, MA). The assay kit consisted of a “9-plex” panel of several cytokines (IFN-α2, IL-8, IL-1β, IL-10, IL-15, IL12_p70_, TNF-α, IFN-ϒ and IL-6). Assays were carried out on a Luminex™ 200 platform.

For IFN-λ and IL-22 detection, a DuoSet ELISA kit containing capture/detection antibodies and recombinant protein standard were purchased from R&D Systems (Minneapolis, MN)

### Statistical analysis

Data are reported as median and range interquartile, unless otherwise indicated. Statistical analysis was performed using R software v. 3.0.2 [17] (with Mann-Whitney for comparison between patients of different groups); *P*-value ≤ 0.05 was considered as the nominal threshold for statistical significance. Non-parametric statistics were used for analysis because the data were not normally distributed.

Cytokine levels in patients from the same group were compared by the Wilcoxon rank-sum test. Spearman’s rank correlation coefficients were used to quantify the association between cytokine concentration and clinical parameters. Bonferroni correction was employed to correct for multiple test.

## Results

### Characteristics of patients and clinical data

A total of 63 children were enrolled in the study, classified in two groups: RV-infected group (*n* = 27) and control group (*n* = 36). RV-infected group age ranged from 1 to 40 months (median of 12 months) at recruitment and from 5 to 47 (median of 18 months) at convalescence. Clinical characteristics of the patients are summarized in **Table 1**. Control group age ranged from 1.8 to 2.3 months (median of 2.1 months) at recruitment.

Salivary cytokine levels were measured in all groups, RV-infected (acute phase), RV-convalescence and control group.

**Table 1:** Summary of main clinical data of rotavirus infected patients included in the study. Data are expressed as mean (SD).

	**RV-Infected** **(acute phase)**	**RV-Convalescence**	**Control group**
	(*n* = 27)	(*n* = 27)	*(n = 36)*
Age (months)	12 (1–40)	18 (5–47)	2.1 (1.8–2.3)
Sex (Male:Female)	16:11	16:11	18:18
Vesikari’s score	11.0 (3.4)	–	–
Length of stay in hospital (days)	5.7 (3.0)	–	–
Temperature (°C)	38.7 (0.6)	–	–
Vomiting episodes per day	3.1 (4.7)	–	–
Duration of vomiting (days)	2.0 (1.9)	–	–
Number of stools per day	4.9 (5.0)	–	–
Duration of diarrhea (days)	4.5 (2.1)	–	–
Level of dehydration	1.6 (1.1)	–	–

### Cytokine profiles in saliva of RV patients

**Fig 1:**
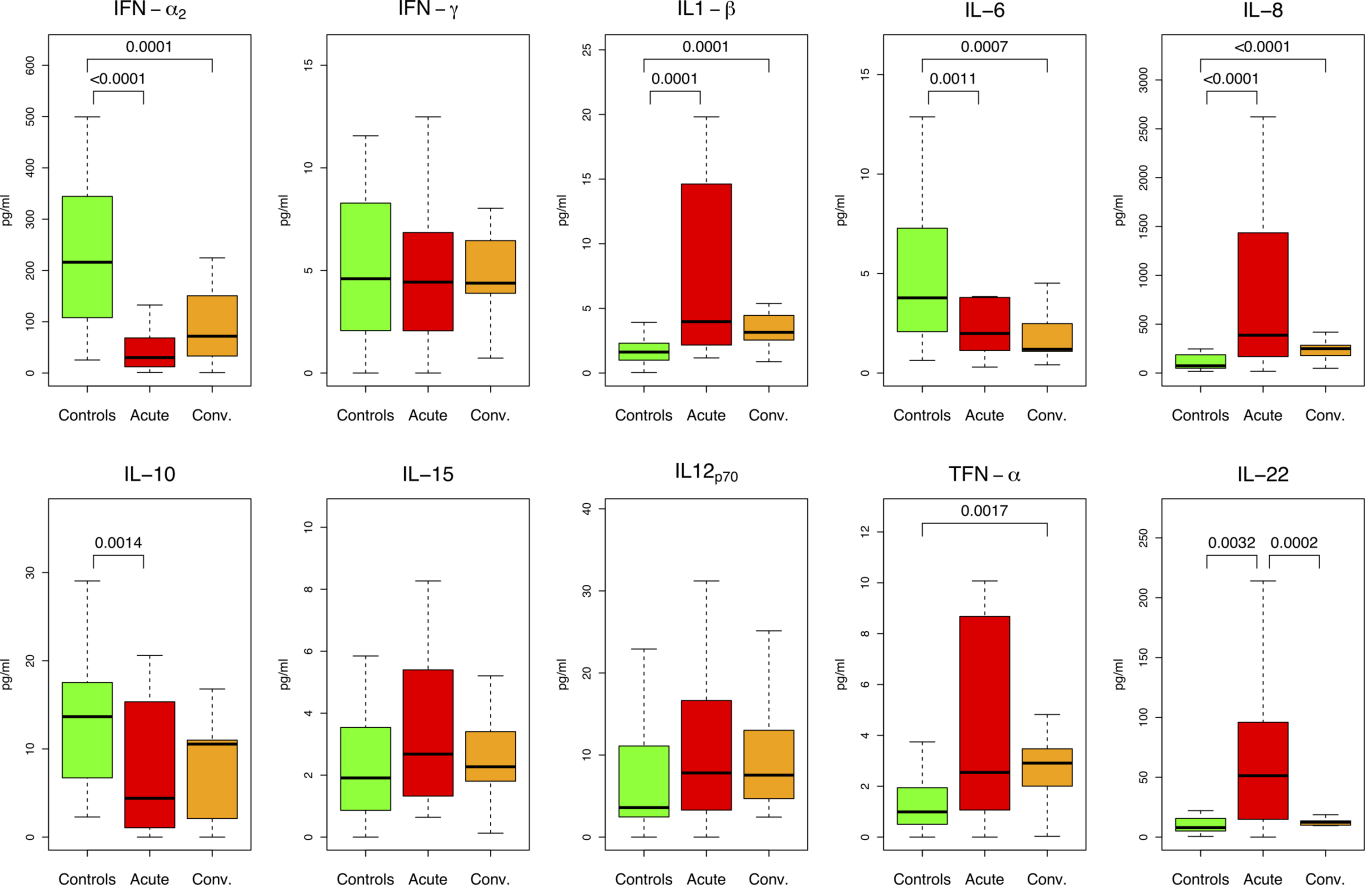
Salivary cytokine concentrations for all groups. Data are represented as median ± interquartile range. Only *P*-values surpassing Bonferroni threshold are indicated.

RV-infected patients in the acute phase showed differences in median levels of IL-22 compared to the convalescence period (**Tables 2 and 3** and **Fig 1**). Levels of IL-22 were found significantly increased (*P*-value < 0.001) in acute-phase samples (47.9 pg/ml) compared with the convalescence period (12.5 pg/ml). All other differences found were not significant after Bonferroni adjustment. We could not detect signal for IFN-λ in any sample of both groups.

**Table 2:** Salivary concentrations of selected cytokines in RV-infected patients (acute phase and at convalescence) and in healthy control children. Data are shown as median (pg/ml) and interquartile ranges (25%-75%) for all groups. nd: not detectable.

	**RV-infected patients**		**Control Group**
**Cytokines**	Acute phase	Convalescence	
**IFN-α**_**2**_	30.1 (12.2–68.5)	71 (31.2–76.8)	216.1 (110.4–337.1)
**IFN-γ**	4.1 (1.9–6.5)	4.3 (3.9–5.2)	4 (2–7.5)
**IL-1β**	3.2 (1.8–4.6)	3 (2.3–3.5)	1.4 (0.9–2)
**IL-6**	1.4 (0.7–2.9)	1.2 (1.1–2.3)	3.6 (2.1–6.8)
**IL-8**	387 (168.4–1435.3)	248.6 (178.8–249.7)	62.6 (48.7–143.9)
**IL-10**	3.8 (1–13)	9 (2.1–10.9)	13 (6.6–17.1)
**IL-15**	2.6 (1.3–5.1)	2.3 (1.6–2.6)	1.7 (0.8–3.1)
**IL12**_**p70**_	7.6 (3.2–15.4)	7.6 (4.3–11.4)	3.2 (2.5–9.4)
**TNF-α**	2 (1–4.4)	2.9 (1.8–3.3)	0.9 (0.5–1.5)
**IL-22**	47.9 (6.8–58.8)	12.5 (11.7–13.5)	7.9 (5.2–15.5)
**IFN-λ**_**1**_	*nd*	*nd*	*nd*

**Table 3:** *P*-values of comparisons between the different groups analyzed in the present study.

**Cytokines**	**AC *vs*. CV**	**AC *vs*. HC**	**CV *vs*. HC**
**IFN-α**_**2**_	0.0547	<0.0001*	0.0001*
**IFN-γ**	0.3038	0.7845	0.908
**IL-1β**	0.1429	0.0001*	0.0001*
**IL-6**	0.0502	0.0011*	0.0007*
**IL-8**	0.0290	<0.0001*	<0.0001*
**IL-10**	0.1907	0.0014*	0.0053
**IL-15**	0.2774	0.0479	0.3517
**IL12**_**p70**_	0.4524	0.0374	0.0245
**TNF-α**	0.1893	0.0053	0.0017*
**IL-22**	0.0002*	0.0032*	0.0769
**IFN-λ**_**1**_	-	-	-

AC: acute patients; CV: convalescence patients; HC: healthy control children;

*: significant after Bonferroni correction.

Saliva from acute-phase infected patients showed significant differences when compared to those measured in for healthy controls for the following cytokines: IFN-x_2_, IL-1β, IL-6, IL-8 and IL-22 (**Tables 2** and **3**, and **Fig 1**).

Finally, IL-1β, IL-6, IFN-α_2_, IL8, and TNF-α were differentially expressed in convalescent *vs*. healthy control children (**Tables 2 and 3, and Fig 1**).

**Fig 2:**
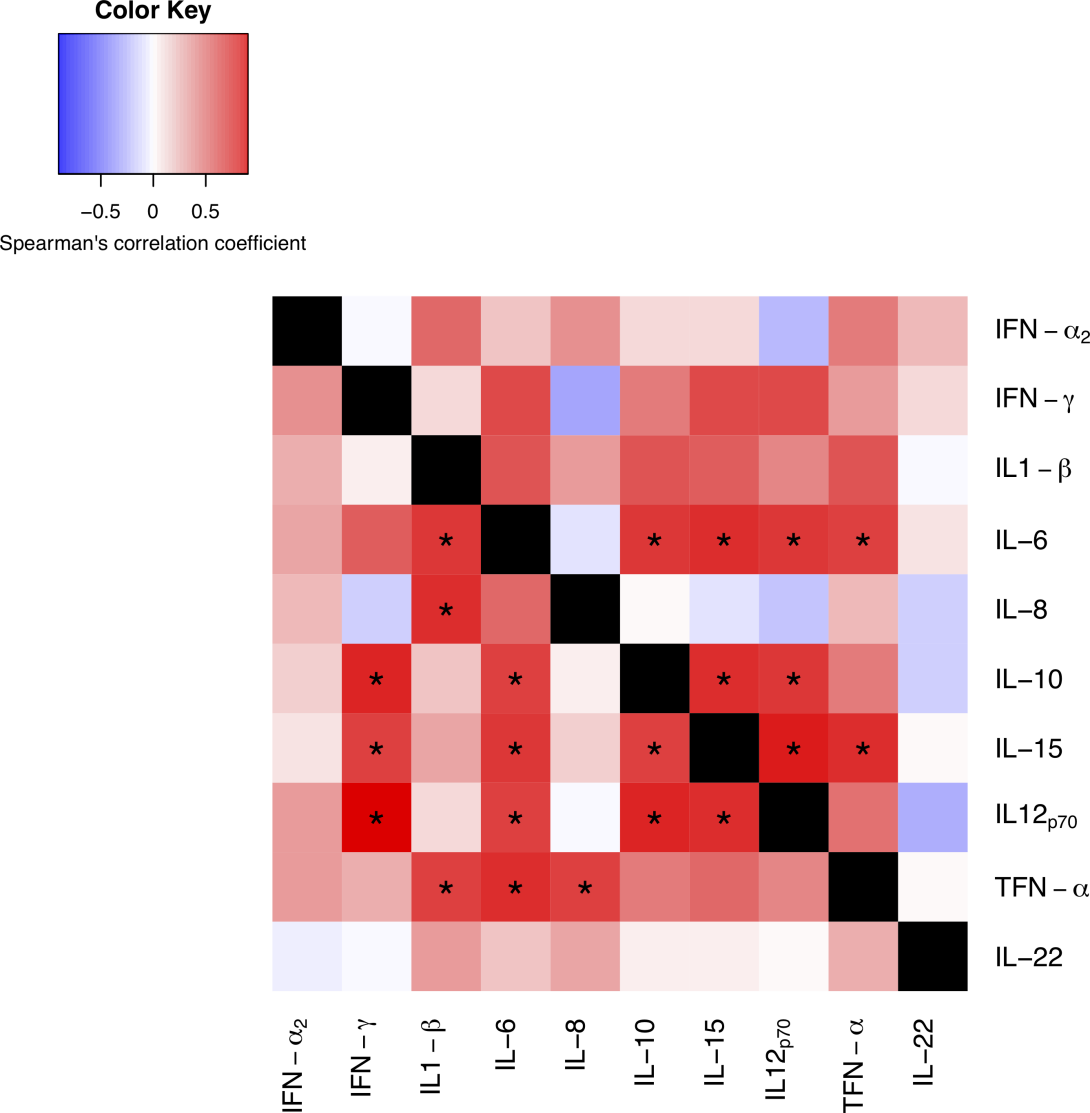
Heatmap of cytokine expression correlations in RV-infected patients (acute-phase vs convalescence). Heatmap of infected patients: below the diagonal are the correlations in acute phase samples, while above the diagonal are the correlations in convalescence samples. Blue and red colors indicate the direction of the correlation as indicated in the legend. Asterisks indicate statistical significant correlations after Bonferroni correction.

Statistically significant correlations between cytokine levels were observed between acute and convalescence patients (**Fig 2 and S1 Table**). As expected, saliva sample from acute-phase patients showed a correlative rise of pro-inflammatory cytokines (e.g. IFN-γ, IL-1β, IL-6, IL-8 and TNF-α). Convalescence saliva samples showed similar significant correlation and rise of pro-inflammatory cytokines, especially at IL-6, IL-10 and IL-15 rise. IL-22, a cytokine of non-epithelial origin did not show correlation with any other cytokines in both groups of samples.

### Salivary cytokine profiles and clinical parameters

We first conducted analyses to examine if salivary cytokine levels in acute-phase infected children were age dependent, to avoid potential interferences in our analysis. There were no significant age-related differences in any cytokine response (**Table 4**), as determined by Spearman’s rank correlation coefficients.

**Table 4:** Correlation coefficients between saliva cytokine levels in RV-infected patients and age, Vesikari’s severity score and length of hospital stay. *P*-value of Spearman’s rank correlation and Pearson’s correlation coefficients for cytokines values are expressed between parentheses.

	**Age (months)**		**Vesikari’s severity score**		**Length of stay (days)**	
**Cytokines**	Spearman’scorrelation	Pearson’s correlation	Spearman’scorrelation	Pearson’scorrelation	Spearman’scorrelation	Pearson’scorrelation
**IFN-α**_**2**_	-0.09 (0.660)	-0.09 (0.67)	-0.09 (0.648)	-0.1 (0.611)	-0.16 (0.419)	0.03 (0.881)
**IFN-γ**	-0.3 (0.132)	-0.17 (0.396)	-0.4 (0.039)	-0.23 (0.249)	-0.04 (0.83)	0.02 (0.925)
**IL-1β**	0.16 (0.436)	0.12 (0.543)	0.09 (0.670)	-0.01 (0.978)	-0.32 (0.109)	-0.15 (0.449)
**IL-6**	-0.28 (0.155)	-0.26 (0.184)	-0.43 (0.026)	-0.25 (0.204)	-0.11 (0.582)	0.44 (0.020)
**IL-8**	0.14 (0.474)	0.18 (0.370)	-0.09 (0.665)	-0.11 (0.571)	-0.1 (0.633)	-0.01 (0.948)
**IL-10**	-0.24 (0.236)	0.02 (0.918)	-0.41 (0.035)	-0.07 (0.712)	-0.08 (0.690)	-0.26 (0.199)
**IL-15**	-0.32 (0.099)	-0.28 (0.153)	-0.31 (0.121)	-0.22 (0.271)	-0.09 (0.658)	-0.06 (0.758)
**IL12**_**p70**_	-0.44 (0.020)	-0.32 (0.101)	-0.42 (0.031)	-0.35 (0.072)	0.03 (0.867)	0.26 (0.192)
**TNF-α**	0.02 (0.916)	0.07 (0.735)	-0.18 (0.374)	-0.03 (0.884)	-0.25 (0.205)	-0.31 (0.111)
**IL-22**	0.37 (0.059)	0.27 (0.179)	0.04 (0.837)	-0.07 (0.729)	-0.26 (0.190)	-0.16 (0.417)
**IFN-λ**_**1**_	-	-	-	-	-	-

We next examined possible associations between the levels of cytokines in acute-phase saliva, and several clinical parameters of disease (**Table 4**). We did not find any cytokine significantly associated to Vesikari’s total severity score, length of hospital stay, or age.

## Discussion

To the best of our knowledge, the results from the present study provide the first evidence for a cytokine-mediated response in oral mucosa in children exposed to natural RV infection. The oral epithelial cells recognize pathogen invaders through their specific innate receptors and stimulate epithelial cells to produce cytokines either by constitutive or inductive pathways [18]. This epithelial cytokine response in oral mucosa had been demonstrated for local inflammatory or infectious events but, it had not been investigated previously for an intestinal infection such as RV.

Rotavirus, the leading cause of severe gastroenteritis in infants and young children around the world, infects preferentially terminally differentiated villous enterocytes of the upper small intestine [19]. Although many case reports show that infection is not limited to the gastrointestinal tract [20] [21] with evidences of systemic transcriptional changes caused by rotavirus infection [22], so far there was no evidence for RV infection of oral mucosa epithelium.

Significant differences in the levels of several cytokines were observed in infected RV-patients, suggesting a systemic response to RV also expressed at the oral mucosa. Our data also show that there is a complex pattern of cytokine co-expression, suggesting that a coordinated immune response associated to a rise of pro-inflammatory cytokines exists. This is particularly remarkable in acute-phase saliva samples, suggesting transference of the inflammatory response from intestine to saliva in a well-orchestrated way.

Acute-phase RV-infected patients showed increased levels of several cytokines, and particularly of IL-22, all of them representative of innate immunity. IL-8 and IL-12, both increased in our patients, are potent pro-inflammatory cytokines and chemo-attractant factors for diverse immune cells such as polymorphonuclear cells and lymphocytes. These cytokines are secreted by the epithelium in response to pathogen entry [23,24]. IFN-γ, a cytokine from the adaptive system that is produced mainly by T-lymphocytes, was not found to be differently expressed in our subjects. IFN-λ_1_, a recently described cytokine that seems to play an important role in the epithelium defense from viral pathogens, was not even detected in our patients. Although there is no evidence of RV entry into oral mucosa epithelial cells, our data suggest that the oral mucosa responds by secreting these pro-inflammatory mediators, presumably through immune communication from intestinal epithelia, showing a global host mucosal immune response to RV infection.

IFN-α_2_ levels were found significantly low in acute-phase infected patients when compared to healthy children. Several studies indicate that the constitutive expression of type I interferon in mucosal surfaces plays an important role in controlling the proliferation and function of the epithelium, and regulating epithelial renewal [25–27]. Rotaviruses have evolved a range of specific mechanisms to evade the type I interferon antiviral response of the host in order to achieve successful replication in the host epithelium [28], for example preventing detection of viral components, or inhibiting the function of transcription factors that initiate interferon response [29]. However, it is unlikely that the low levels of IFN-α observed in oral mucosa of infected patients are due to specific inhibition promoted by RV, since there is no evidence for entry of virus in oral epithelia. It seems more likely that intestinal mucosa injury interferes with the normal homeostasis of interferon production in the whole mucosa.

High levels of IL-22, a cytokine not associated with an epithelial origin, were found in oral mucosa of acute-phase RV-infected patients. This cytokine belongs to the superfamily of IL-10 [30] and is mainly synthetized by innate lymphoid cells (ILC3), dendritic cells and natural killer (NK) cells [31]. There is no evidence of IL-22 synthesis by epithelial cells; therefore, the presence of this cytokine in oral mucosa is most likely associated with a lymphoid innate immune response, suggesting presence of activated lymphoid cells in oral mucosa in response to intestinal RV infection. A major target for this cytokine are epithelial cells (respiratory and gut) where it acts as a potent mediator of cellular inflammatory process against invader pathogens [32]. Administration of IL-22 and IL-18 in intestinal epithelial cells in mice, produced elimination of RV infection acting as a broad-spectrum antiviral agent [33]. Also, IL-22 has been related to protective functions in epithelia through a regenerative action on injured epithelia after infection [32,34,35]. Salivary levels of IL-22 show a significant correlation with clinical parameters of Sjögren’s disease, indicating a critical role for this cytokine in the pathogenesis of this autoimmune disease that targets salivary glands [36]. The finding of elevated levels of IL-22 in oral mucosa shows that not only epithelial cells respond to the intestinal RV infection producing pro-inflammatory signals, but also mucosal lymphoid innate cells are involved in this response.

Several clinical parameters were investigated with regards to cytokine levels, although none of them reached statistical significance. IFN-γ, IL-6, IL-10 and IL12_p70_ were statistically correlated with Vesikari’s severity score, but significance did not surpass multiple test correction (**Table 4**). Whether this reflects just a lack of association or insufficient statistical power to detect them can only be addressed through more powerful cohort studies.

Besides limited sample size, age differences between children from RV-infected (acute-phase and convalescence) and healthy control groups might constitute a limitation to our study. However, we did not find any link between age and cytokine levels in saliva samples in either our samples or the literature, even for sera cytokine levels. [37]

In conclusion, our data suggest the existence of an oral mucosa cytokine host response to RV infection. Several pro-inflammatory innate cytokines produced by epithelial cells, such as IL-8, IL-1β, IL-12 and TNF-α, were elevated in saliva after infection. In addition, production of IL-22, presumably from mucosa innate lymphoid circulating cells, was elevated. IFN-α_2_ was inhibited probably as consequence of RV homeostasis disruption.

Although the present study needs further validation, our preliminary data indicate that RV infection is not confined to the intestinal mucosa, suggesting a whole mucosal implication as a result of the integrative mucosal immune response.

## Supporting information

S1 Table Correlation coefficients between saliva cytokine levels in RV-infected subjects at baseline and at convalescence. Spearman’s correlations rank coefficient for each pair of cytokine values (P-value between parentheses) for RV-infected patients. Correlations for cytokines measured in acute-phase are shown below the diagonal; correlations for cytokines measured in convalescent- phase are shown above the diagonal.
(DOCX)

## References

[1] Ganz T (2002) Epithelia: not just physical barriers. Proc Natl Acad Sci U S A 99: 3357–3358. doi: 10.1073/pnas.072073199 11904396 10.1073/pnas.072073199 PMC122525

[2] Sugawara S, Uehara A, Tamai R, Takada H (2002) Innate immune responses in oral mucosa. J Endotoxin Res 8: 465–468. doi: 10.1179/096805102125001082 12697091 10.1179/096805102125001082

[3] Yin L, Dale BA (2007) Activation of protective responses in oral epithelial cells by Fusobacterium nucleatum and human beta-defensin-2. J Med Microbiol 56: 976–987. doi: 10.1099/jmm.0.47198-0 17577065 10.1099/jmm.0.47198-0

[4] Stadnyk AW (1994) Cytokine production by epithelial cells. FASEB J 8: 1041–1047. 7926369 10.1096/fasebj.8.13.7926369

[5] Stadnyk AW (2002) Intestinal epithelial cells as a source of inflammatory cytokines and chemokines. Can J Gastroenterol 16: 241–246. 11981577 10.1155/2002/941087

[6] Aase A, Sommerfelt H, Petersen LB, Bolstad M, Cox RJ, Langeland N et al. (2015) Salivary IgA from the sublingual compartment as a novel noninvasive proxy for intestinal immune induction. Mucosal Immunol. 10.1038/mi.2015.107 26509875

[7] Macpherson AJ, Slack E (2007) The functional interactions of commensal bacteria with intestinal secretory IgA. Curr Opin Gastroenterol 23: 673–678. doi: 10.1097/MOG.0b013e3282f0d012 17906446 10.1097/MOG.0b013e3282f0d012

[8] McGhee JR, Fujihashi K (2012) Inside the mucosal immune system. PLoS Biol 10: e1001397 doi: 10.1371/journal.pbio.1001397 23049482 10.1371/journal.pbio.1001397 PMC3457930

[9] Craig SW, Cebra JJ (1971) Peyer's patches: an enriched source of precursors for IgA-producing immunocytes in the rabbit. J Exp Med 134: 188–200. 4934147 10.1084/jem.134.1.188 PMC2139023

[10] Pierce NF, Gowans JL (1975) Cellular kinetics of the intestinal immune response to cholera toxoid in rats. J Exp Med 142: 1550–1563. 1238506 10.1084/jem.142.6.1550 PMC2190063

[11] Brandtzaeg P, Johansen FE, Baekkevold ES, Carlsen HS, Farstad IN (2004) The traffic of mucosal lymphocytes to extraintestinal sites. J Pediatr Gastroenterol Nutr 39 Suppl 3: S725–726. 15167361 10.1097/00005176-200406003-00004

[12] Macpherson AJ, McCoy KD, Johansen FE, Brandtzaeg P (2008) The immune geography of IgA induction and function. Mucosal Immunol 1: 11–22. doi: 10.1038/mi.2007.6 19079156 10.1038/mi.2007.6

[13] Sandros J, Karlsson C, Lappin DF, Madianos PN, Kinane DF and Papapanou PN (2000) Cytokine responses of oral epithelial cells to Porphyromonas gingivalis infection. J Dent Res 79: 1808–1814. doi: 10.1177/00220345000790101301 11077999 10.1177/00220345000790101301

[14] Deo V, Bhongade ML (2010) Pathogenesis of periodontitis: role of cytokines in host response. Dent Today 29: 60–62, 64–66; quiz 68–69. 20973418

[15] Silva N, Abusleme L, Bravo D, Dutzan N, Garcia-Sesnich J, Vernal R et al (2015) Host response mechanisms in periodontal diseases. J Appl Oral Sci 23: 329–355. doi: 10.1590/1678-775720140259 26221929 10.1590/1678-775720140259 PMC4510669

[16] Roescher N, Tak PP, Illei GG (2010) Cytokines in Sjogren's syndrome: potential therapeutic targets. Ann Rheum Dis 69: 945–948. doi: 10.1136/ard.2009.115378 20410069 10.1136/ard.2009.115378 PMC3044243

[17] Team RDC (2011) R: A Language and environment for statistical computing: R Foundation for Statistical Computing.

[18] Steele C, Fidel PL Jr. (2002) Cytokine and chemokine production by human oral and vaginal epithelial cells in response to Candida albicans. Infect Immun 70: 577–583. doi: 10.1128/IAI.70.2.577-583.2002 11796585 10.1128/IAI.70.2.577-583.2002 PMC127706

[19] Osborne MP, Haddon SJ, Spencer AJ, Collins J, Starkey WG, Wallis TS et al (1988) An electron microscopic investigation of time-related changes in the intestine of neonatal mice infected with murine rotavirus. J Pediatr Gastroenterol Nutr 7: 236–248. 2832583 10.1097/00005176-198803000-00014

[20] Rivero-Calle I, Gomez-Rial J, Martinon-Torres F (2016) Systemic features of rotavirus infection. J Infect 72 Suppl: S98–S105. doi: 10.1016/j.jinf.2016.04.029 27181101 10.1016/j.jinf.2016.04.029

[21] Candy DC (2007) Rotavirus infection: a systemic illness? PLoS Med 4: e117 doi: 10.1371/journal.pmed.0040117 17439293 10.1371/journal.pmed.0040117 PMC1852121

[22] Salas A, Marco-Puche G, Trivino JC, Gomez-Carballa A, Cebey-Lopez M, Rivero-Calle I et al (2016) Strong down-regulation of glycophorin genes: A host defense mechanism against rotavirus infection. Infect Genet Evol 44: 403–411. doi: 10.1016/j.meegid.2016.07.044 27491455 10.1016/j.meegid.2016.07.044

[23] Eckmann L, Kagnoff MF, Fierer J (1993) Epithelial cells secrete the chemokine interleukin-8 in response to bacterial entry. Infect Immun 61: 4569–4574. 8406853 10.1128/iai.61.11.4569-4574.1993 PMC281206

[24] Waterhouse CC, Stadnyk AW (1999) Rapid expression of IL-1beta by intestinal epithelial cells in vitro. Cell Immunol 193: 1–8. doi: 10.1006/cimm.1999.1468 10202107 10.1006/cimm.1999.1468

[25] Katlinskaya YV, Katlinski KV, Lasri A, Li N, Beiting DP, Durham AC et al (2016) Type I Interferons Control Proliferation and Function of the Intestinal Epithelium. Mol Cell Biol 36: 1124–1135. doi: 10.1128/MCB.00988-15 26811327 10.1128/MCB.00988-15 PMC4800802

[26] Bielenberg DR, Fidler IJ, Bucana CD (1998) Constitutive expression of interferon beta in differentiated epithelial cells exposed to environmental stimuli. Cancer Biother Radiopharm 13: 375–382. doi: 10.1089/cbr.1998.13.375 10851428 10.1089/cbr.1998.13.375

[27] Hsu AC, Parsons K, Barr I, Lowther S, Middleton D, Hansbro PM et al (2012) Critical role of constitutive type I interferon response in bronchial epithelial cell to influenza infection. PLoS One 7: e32947 doi: 10.1371/journal.pone.0032947 22396801 10.1371/journal.pone.0032947 PMC3292582

[28] Sherry B (2009) Rotavirus and reovirus modulation of the interferon response. J Interferon Cytokine Res 29: 559–567. doi: 10.1089/jir.2009.0072 19694545 10.1089/jir.2009.0072 PMC2956631

[29] Arnold MM, Sen A, Greenberg HB, Patton JT (2013) The battle between rotavirus and its host for control of the interferon signaling pathway. PLoS Pathog 9: e1003064 doi: 10.1371/journal.ppat.1003064 23359266 10.1371/journal.ppat.1003064 PMC3554623

[30] Pestka S, Krause CD, Sarkar D, Walter MR, Shi Y and Fisher PB (2004) Interleukin-10 and related cytokines and receptors. Annu Rev Immunol 22: 929–979. doi: 10.1146/annurev.immunol.22.012703.104622 15032600 10.1146/annurev.immunol.22.012703.104622

[31] Takatori H, Kanno Y, Watford WT, Tato CM, Weiss G, Ivanov II et al (2009) Lymphoid tissue inducer-like cells are an innate source of IL-17 and IL-22. J Exp Med 206: 35–41. doi: 10.1084/jem.20072713 19114665 10.1084/jem.20072713 PMC2626689

[32] Zenewicz LA, Flavell RA (2011) Recent advances in IL-22 biology. Int Immunol 23: 159–163. doi: 10.1093/intimm/dxr001 21393631 10.1093/intimm/dxr001

[33] Zhang B, Chassaing B, Shi Z, Uchiyama R, Zhang Z, Denning TL et al (2014) Viral infection. Prevention and cure of rotavirus infection via TLR5/NLRC4-mediated production of IL-22 and IL-18. Science 346: 861–865. doi: 10.1126/science.1256999 25395539 10.1126/science.1256999 PMC4788408

[34] Kumar P, Thakar MS, Ouyang W, Malarkannan S (2013) IL-22 from conventional NK cells is epithelial regenerative and inflammation protective during influenza infection. Mucosal Immunol 6: 69–82. doi: 10.1038/mi.2012.49 22739232 10.1038/mi.2012.49 PMC3835350

[35] Lindemans CA, Calafiore M, Mertelsmann AM, O'Connor MH, Dudakov JA, Jeng RR et al (2015) Interleukin-22 promotes intestinal-stem-cell-mediated epithelial regeneration. Nature 528: 560–564. doi: 10.1038/nature16460 26649819 10.1038/nature16460 PMC4720437

[36] Lavoie TN, Stewart CM, Berg KM, Li Y, Nguyen CQ (2011) Expression of interleukin-22 in Sjogren's syndrome: significant correlation with disease parameters. Scand J Immunol 74: 377–382. doi: 10.1111/j.1365-3083.2011.02583.x 21645026 10.1111/j.1365-3083.2011.02583.x PMC3250060

[37] Sack U, Burkhardt U, Borte M, Schadlich H, Berg K and Emmrich F (1998) Age-dependent levels of select immunological mediators in sera of healthy children. Clin Diagn Lab Immunol 5: 28–32. 9455875 10.1128/cdli.5.1.28-32.1998 PMC121386

