# Supplementary material for: Rotavirus intestinal infection induces an oral mucosa cytokine response
Source: PLoS One. 2018 Apr 5;13(4):e0195314. doi: 10.1371/journal.pone.0195314 (PMC5886481; doi:10.1371/journal.pone.0195314)
Supplement: S1 Table — Spearman’s correlations rank coefficient for each pair of cytokine values (P-value between parentheses) for RV-infected patients. Correlations for cytokines measured in acute-phase are shown below the diagonal; correlations for cytokines measured in convalescent- phase are shown above the diagonal. (DOCX) [file pone.0195314.s001.docx]

|  | **IFN α_2_** | **IFN-γ** | **IL-1β** | **IL 6** | **IL-8** | **IL-10** | **IL-15** | **IL12p70** | **TNF-α** | **IL-22** |
| --- | --- | --- | --- | --- | --- | --- | --- | --- | --- | --- |
| **IFN α_2_** |  | -0.03 (0.902) | 0.52 (0.015) | 0.19 (0.402) | 0.38 (0.086) | 0.11 (0.627) | 0.13 (0.561) | -0.22 (0.336) | 0.44 (0.046) | 0.25 (0.282) |
| **IFN-γ** | 0.37 (0.058) |  | 0.13 (0.580) | 0.61 (0.003) | -0.32 (0.160) | 0.45 (0.042) | 0.61 (0.003) | 0.63 (0.002) | 0.34 (0.130) | 0.13 (0.565) |
| **IL-1β** | 0.28 (0.153) | 0.04 (0.842) |  | 0.58 (0.005) | 0.36 (0.112) | 0.60 (0.004) | 0.55 (0.009) | 0.41 (0.063) | 0.6 (0.004) | -0.01 (0.972) |
| **IL 6** | 0.31 (0.117) | 0.55 (0.003) | 0.7 (<0.001*) |  | -0.10 (0.668) | 0.72 (<0.001*) | 0.75 (<0.001*) | 0.71 (<0.001*) | 0.66 (0.001*) | 0.08 (0.716) |
| **IL-8** | 0.23 (0.243) | -0.17 (0.392) | 0.75 (<0.001*) | 0.51 (0.007) |  | 0.02 (0.918) | -0.09 (0.693) | -0.19 (0.414) | 0.24 (0.295) | -0.15 (0.514) |
| **IL-10** | 0.17 (0.406) | 0.77 (<0.001*) | 0.21 (0.298) | 0.67 (<0.001*) | 0.06 (0.765) |  | 0.72 (<0.001*) | 0.7 (<0.001*) | 0.46 (0.035) | -0.15 (0.523) |
| **IL-15** | 0.11 (0.598) | 0.66 (<0.001*) | 0.32 (0.107) | 0.72 (<0.001*) | 0.16 (0.435) | 0.65 (<0.001*) |  | 0.81 (<0.001*) | 0.74 (<0.001*) | 0.02 (0.940) |
| **IL12_p70_** | 0.33 (0.091) | 0.90 (<0.001*) | 0.14 (0.475) | 0.65 (<0.001*) | -0.03 (0.886) | 0.76 (<0.001*) | 0.74 (<0.001*) |  | 0.48 (0.028) | -0.26 (0.253) |
| **TNF-α** | 0.33 (0.089) | 0.28 (0.165) | 0.65 (<0.001*) | 0.74 (<0.001*) | 0.68 (<0.001*) | 0.46 (0.015) | 0.54 (0.004) | 0.4 (0.039) |  | 0.03 (0.900) |
| **IL-22** | -0.04 (0.843) | -0.01 (0.964) | 0.34 (0.081) | 0.2 (0.313) | 0.32 (0.100) | 0.04 (0.826) | 0.06 (0.772) | 0.01 (0.962) | 0.25 (0.202) |  |

**S1 Table** . Correlation coefficients between saliva cytokine levels in RV-infected subjects at baseline and at convalescence. Spearman’s correlations rank coefficient for each pair of cytokine values (*P*-value between parentheses) for RV-infected patients. Correlations for cytokines measured in acute-phase are shown below the diagonal; correlations for cytokines measured in convalescent- phase are shown above the diagonal.
